# Increased Global and Local Efficiency of Human Brain Anatomical Networks Detected with FLAIR-DTI Compared to Non-FLAIR-DTI

**DOI:** 10.1371/journal.pone.0071229

**Published:** 2013-08-13

**Authors:** Shumei Li, Bin Wang, Pengfei Xu, Qixiang Lin, Gaolang Gong, Xiaoling Peng, Yuanyuan Fan, Yong He, Ruiwang Huang

**Affiliations:** 1 Center for the Study of Applied Psychology, Key Laboratory of Mental Health and Cognitive Science of Guangdong Province, School of Psychology, South China Normal University, Guangzhou, P. R. China; 2 State Key Laboratory of Cognitive Neuroscience and Learning, Beijing Normal University, Beijing, P. R. China; Banner Alzheimer’s Institute, United States of America

## Abstract

Diffusion-weighted MRI (DW-MRI), the only non-invasive technique for probing human brain white matter structures *in vivo*, has been widely used in both fundamental studies and clinical applications. Many studies have utilized diffusion tensor imaging (DTI) and tractography approaches to explore the topological properties of human brain anatomical networks by using the single tensor model, the basic model to quantify DTI indices and tractography. However, the conventional DTI technique does not take into account contamination by the cerebrospinal fluid (CSF), which has been known to affect the estimated DTI measures and tractography in the single tensor model. Previous studies have shown that the Fluid-Attenuated Inversion Recovery (FLAIR) technique can suppress the contribution of the CSF to the DW-MRI signal. We acquired DTI datasets from twenty-two subjects using both FLAIR-DTI and conventional DTI (non-FLAIR-DTI) techniques, constructed brain anatomical networks using deterministic tractography, and compared the topological properties of the anatomical networks derived from the two types of DTI techniques. Although the brain anatomical networks derived from both types of DTI datasets showed small-world properties, we found that the brain anatomical networks derived from the FLAIR-DTI showed significantly increased global and local network efficiency compared with those derived from the conventional DTI. The increases in the network regional topological properties derived from the FLAIR-DTI technique were observed in CSF-filled regions, including the postcentral gyrus, periventricular regions, inferior frontal and temporal gyri, and regions in the visual cortex. Because brain anatomical networks derived from conventional DTI datasets with tractography have been widely used in many studies, our findings may have important implications for studying human brain anatomical networks derived from DW-MRI data and tractography.

## Introduction

Diffusion-weighted magnetic resonance imaging (DW-MRI) is the only available tool for non-invasively probing human brain tissue microstructure and the microanatomical organization of human brain white matter *in vivo*. Recent advances in diffusion tensor imaging (DTI) techniques and white matter tractography have made it possible to visualize the fiber tracts comprised of coherently oriented axons and to map the anatomical connectivity patterns of healthy and diseased human brains [1], [2], [3], [4]. In the last few years an explosion of studies that constructed human brain anatomical networks using DTI techniques and tractography and analyzed the topological characteristics of the brain networks using graph theory has occurred [5], [6], [7], [8], [9], [10]. The analysis of human brain anatomical networks based on graph theory has been applied to study normal aging [6], behavior performance [11], and various brain disorders [12], [13], [14], [15]. In order to investigate the reliability and replicability of human brain anatomical networks based on graph theory, several studies [10], [16] have investigated the influence of different scanning techniques or different definitions of nodes on the topological properties of anatomical networks.Acquiring reliable DTI datasets is undoubtedly essential if we want to obtain accurate information from DTI and tractography for understanding the properties of human brain anatomical networks [17], [18]. In order to construct human brain anatomical networks, we need to define nodes that represent brain regions and edges (or links) that represent the strength of the connectivity between the nodes. The connectivity strengths are usually estimated using tractography. The basic model used in quantifying DTI indices and tractography is the single tensor model. However, a potential problem which affects the accuracy of tractography and the estimated DTI metrics derived from the single tensor model, but which is often ignored, is contamination by cerebrospinal fluid (CSF) [19], [20], [21], [22], [23]. Previous studies [17], [19] have shown that the single tensor model would lead to highly variable and inaccurate measurements of diffusion when two or more distinct tissues with different diffusion tensors occupy the same voxel. If a voxel contain CSF and brain tissue, the accuracy of its diffusion tensor measurement and the diffusion parameter of the brain tissue estimated using the single tensor model may be significantly influenced [17], [18], [19], [24]. Previous studies showed that CSF contamination can significantly influence diffusion measurements in ways such as overestimating the apparent diffusion coefficient (ADC) by about 15–30% [21] and underestimating the diffusion anisotropy [17], [25], [26], [27].

To reduce the influence of CSF on the measurement of diffusivity and to increase the accuracy of the tractography, at least two kinds of solutions have been proposed to mitigate any CSF contamination. One is to improve the DTI sequence by suppressing the CSF signal using a radio-frequency pulse inversion recovery during DTI acquisition, such as the Fluid-Attenuated Inversion Recovery (FLAIR) technique [27], [28], [29], [30]. Another is to introduce a mathematical model, such as the two compartment tensor model [31], [32], [33], to process the DTI dataset. In the present study, we mainly focus on the effect of the FLAIR-DTI technique on removing the CSF contamination in the single tensor model. Magnetic resonance theory indicates that using the FLAIR-DTI technique to suppress the contamination by the CSF will yield MR signals that primarily represent the contributions of brain tissues (white matter and gray matter). The main reason is that FLAIR suppressed the CSF contamination to DTI signal, and then the measurements of diffusion derived from the single tensor model are more accurate than that obtained from the conventional DTI technique. Although the FLAIR-DTI technique may also diminish the signal-to-noise ratio (SNR) of DTI datasets [34], [35], research indicates that the increased volume of fiber tracts derived when using FLAIR-DTI is primarily due to eliminating CSF effects rather than to a decreased SNR [25]. Therefore, measures of diffusivity and tractography using the single tensor model derived from a FLAIR-DTI dataset should more accurately reflect neural connectivity than those derived from a conventional DTI dataset. To improve the accuracy of the tractography, some studies of microanatomical changes in white matter have already utilized the FLAIR-DTI technique [36], [37].

Even though measures of diffusivity and tractography derived from the FLAIR-DTI technique should be more useful for analyzing the property of brain tissue and brain connectivity, no study to date has investigated the differences between human brain anatomical networks derived from FLAIR-DTI and those derived using conventional DTI (non-FLAIR-DTI) techniques. In this study, we acquired DTI datasets from twenty-two normal, healthy volunteers using both FLAIR-DTI and conventional DTI sequences, constructed anatomical networks using a deterministic tractography method, analyzed the topological properties of the networks, and compared the statistical differences between the anatomical networks constructed from the two types of DTI datasets. We hypothesized that the brain anatomical networks derived from the FLAIR-DTI datasets would have more fibers as edges because this method can capture the “real” anatomical connectivity patterns of the human brain due to the reduced CSF contamination. We expected that the brain anatomical networks constructed from the FLAIR-DTI datasets would exhibit more efficient small-world properties and that the topological properties of brain regions with high concentrations of CSF would show significant differences from those constructed using the conventional DTI dataset.

## Materials and Methods

### Subjects

Twenty-two right-handed healthy volunteers (12 F/10 M, aged 18–29 yrs, mean ± *SD* = 20.05±2.73 yrs) participated in this study. None of the volunteers had a history of neurological or psychiatric disease or brain injury. The protocols were approved by the Review Board of the Institute of Cognitive Neuroscience and Learning at Beijing Normal University (BNU). Informed written consent was obtained from each participant prior to the MR scanning.

### MRI Data Acquisition

Each of the twenty-two subjects was scanned on a 3T Siemens Trio MR scanner using a conventional DTI sequence and a FLAIR-DTI sequence with a twelve-channel phased array head coil with the implementation of the parallel imaging scheme GRAPPA (GeneRalized Autocalibrating Partially Parallel Acquisitions) and an acceleration factor of 2. Both the conventional DTI and the FLAIR-DTI scans were performed using a single-shot twice-refocused spin-echo diffusion-weighted EPI sequence [38], with the exception that a slice selective 180° inversion RF-pulse was added before the 90° excitation RF-pulse in the FLAIR-DTI sequence. The parameters of the conventional DTI sequence were as follows: repetition time (TR) = 9000 ms, echo time (TE) = 92 ms, slice thickness = 2 mm, voxel size = 2×2×2 mm^3^, field of view (FOV) = 256 mm×248 mm and data matrix = 128×124, 30 directions with *b* = 1000 s/mm^2^ and a *b* = 0 volume, and 68 transverse slices without gap covering the whole brain. When we acquired the conventional DTI and FLAIR-DTI datasets, we arranged the 30 diffusion-sensitive gradient directions in an icosahedral scheme. The parameters of the FLAIR-DTI sequence were same as those of the conventional DTI sequence except the inversion time (TI) = 2250 ms, and TR = 18000 ms.

In addition, we also acquired 3D brain anatomical images using a T1-weighted MP-RAGE sequence for each subject (TE/TR/TI = 3.44 ms/1900 ms/900 ms, flip angle = 9°, voxel size = 1×1×1 mm^3^, 176 sagittal slices).

All of the MR scans were performed in the same MR scanner in the State Key Laboratory of Cognitive Neuroscience and Learning, Beijing Normal University. For each subject, the scan times for the FLAIR-DTI, the conventional DTI, and the 3D anatomical images were about 10, 5, and 5 minutes, respectively. We scanned each subject twice for both the conventional DTI and FLAIR-DTI sequences. The two DTI datasets from each type of DTI sequence were averaged for each subject in order to improve the signal-to-noise ratio (SNR) before further processing.

### SNR Calculation

Considering the fact that FLAIR-DTI yields lower signal-to-noise ratio (SNR) in the acquired raw images than those in the Conventional-DTI, we calculated the SNR values in each DTI dataset and adjusted its effect to explore the pure CSF effect on the properties of brain anatomical networks. SNR calculations were performed using the most commonly used “two region” method (SNRmean) described by Dietrich [39]. This method uses two regions of interest (ROIs) in a single image, one in the tissue of interest (ROItissue), the other in the image background (ROIair). The SNR was then calculated as the mean value of the signal in the ROItissue divided by the mean value of the signal in the ROIair. Finally, a correction factor (0.8) was used because of the Rayleigh distribution of background noise in magnitude images. In our study, we selected the splenium of the corpus callosum as the ROItissue in the b0 image and calculated the SNR for the conventional DTI datasets (22.078±1.953) and the FLAIR-DTI datasets (20.122±1.510). The SNR value for each subject is listed in Table S1 (Supplementary Materials). Although SNR effect is one of the factors that can affect DTI tractography reliability [40], [41], we should note that the SNRs in the splenium of the corpus callosum calculated here are not a direct measure of the tractography reliability.

### Construction of Human Brain Anatomical Networks

**Figure 1:**
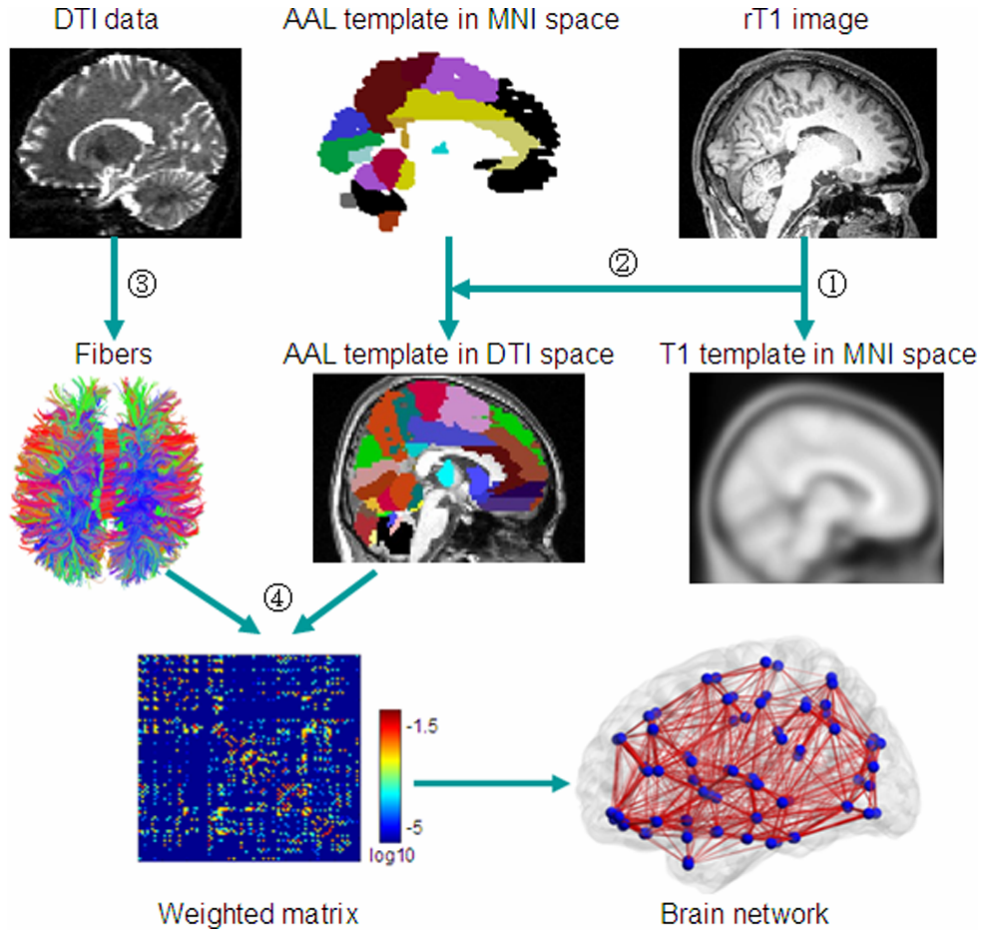
Flowchart for constructing human brain anatomical networks using DTI datasets and tractography. (1) Individual anatomical images were first coregistered into b0 images to obtain rT1 images in diffusion space. The rT1 images were then mapped to a T1-weighted template of ICBM152 in MNI space. (2) The obtained inverse matrix was used to transform the AAL template from the MNI space into individual diffusion space. (3) Fibers in the whole brain were reconstructed using the deterministic tractographic method (DtiStudio software). For display purposes only, fibers shown here were calculated using TrackVis software. (4) Construction of the weighted connectivity matrices and the human brain anatomical networks.

We followed the procedures described in previous studies [5], [42] to construct human brain anatomical networks (Fig. 1). Specifically, the method contains four steps.

#### Step 1: Node definition

The nodes were defined according to the automated anatomical labeling (AAL) template [43] which parcellates the whole cerebral cortex into 90 cortical and subcortical regions. The name and the abbreviations of these ROIs are listed in Table S2 (Supplementary Materials). After performing coregistration, we obtained 90 ROIs in the diffusion space for each subject. Each ROI was defined as a node in a brain network [5], [42].

#### Step 2: Fiber tracking

The white matter fibers of the whole brain for each subject were reconstructed using the DtiStudio software package (Version 3.0.3) [44]. Taking the conventional DTI datasets from one subject as an example, we concatenated all the diffusion images of the two repeated DTI scans (31×2 volumes) and corrected for the effects of head motion and eddy currents by selecting the first *b = *0 image as the reference volume and using an affine registration from the FSL-FDT Toolbox (http://www.fmrib.ox.ac.uk/fsl). The corrected DTI datasets were then split and averaged to generate the averaged 31 volumes of DTI datasets that were subsequently used in fiber tracking. We reconstructed the fibers for the whole brain based on the Fiber Assignment by Continuous Tracking (FACT) algorithm, which was implemented in DtiStudio. Fiber tracking was terminated at voxels with a fractional anisotropy (FA)<0.20 and a tract turning-angle>45°.

#### Step 3: Edge definition

The edges of the anatomical networks were defined as the anatomical connections between any pair of nodes. Given two ROIs 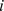 and 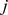, we assumed they were connected if there was at least one fiber 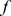 with end points located in these two regions. We calculated the connection density between these two regions as the weighted index 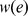 of the edge [45]

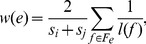
(1)where 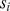 and 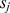 are the cortical surface areas of two ROIs 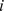 and 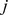, 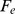 stands for all fibers connecting the two ROIs 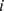 and 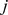, and 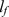 represents the length of the fiber 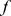.

#### Step 4: Network construction

Taking each ROI as a node and the weighted index between any pair of nodes as the edge, we obtained a weighted brain anatomical network for each subject for each type of DTI data.

### Graph Theoretical Analysis

We used seven global network parameters, the weighted clustering coefficient (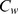), weighted characteristic shortest path length (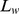), normalized weighted clustering coefficient (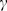), normalized weighted characteristic shortest path length (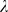), global efficiency (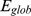), local efficiency (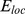), and sparsity (*S*), to characterize the global topological properties of the brain networks. Their definitions are provided in the Text S1 (Supplementary materials). To examine the small-world properties, we computed the normalized clustering coefficient (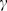) and the normalized characteristic shortest path length (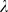). A network is considered as small-world if it satisfies the following criteria [46]: 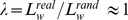 and 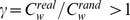, where 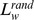 and 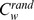 are the mean clustering coefficient and characteristic path length of the matched random networks that keep the same number of nodes, edges, and degree distribution as the real networks [47].

We used four nodal parameters, degree (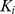), nodal local efficiency (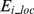), nodal global efficiency (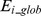), and node betweenness (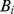) to characterize the nodal properties of the human brain anatomical networks. Their definitions are provided in the Text S1 (Supplementary materials).

### Hub Identification

The hub regions of the brain anatomical networks were identified according to the normalized betweenness, 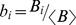, where 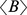 is the average nodal betweenness of the networks. A node 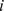 is recognized as a hub region if the value of 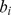 is at least one standard deviation (*SD*) greater than the average normalized betweenness of the network, or 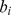
>mean+*SD* [5].

### Backbone Network

Following the methodology used in previous studies [5], [48], [49], we also calculated the backbone network for both types of DTI datasets. Backbone networks are population-based networks that capture an underlying consistent connectivity pattern, rather than being a subject-specific or a very detailed network based on an individual brain. We constructed 90×90 symmetric weighted connectivity matrices by computing the weighted index for each pair of the 90 regions. Then a nonparametric one-tailed sign test (with Bonferroni correction) was applied element-by-element to these weighted connectivity matrices across the subjects to identify the consistent inter-regional anatomical connections (the backbone network).

### Statistical Analysis

To determine whether the CSF had significant effects on the topological properties of human brain anatomical networks formed using the FLAIR-DTI and conventional DTI datasets, a multiple linear regression analysis with a paired *t*-test was performed on each network metric. The effect of SNR was adjusted for all of these analyses. Several previous studies [10], [14], [50], [51], [52], [53] have used the linear regression to model effects of total brain volume, head motion, gender, age and years of education on each network metric for each participant. In this study, we adopted the similar procedure to regress out the influence of SNR on the network metric. We performed the normality test of the residuals on each network metric and found the residuals satisfy the normal distribution. The results of the normality test are listed in Tables S3 and S5, Figures S1 and S2 (Supplementary Materials). For the global and nodal parameters, we used the threshold *p* = 0.05. To solve the problem of multiple comparisons, a height statistical standard (FDR correction, *q* = 0.05) [54] and an extent threshold of *p*<0.01 (uncorrected) was adopted. To justify that the sample size in this study is enough to infer a statistically significant difference, we performed a power analysis according to a previous study [55]. The results of the power analysis are listed in Tables S3 and S4 (Supplementary Materials). The details of normality test and power analysis are provided in Text S2 (Supplementary Materials). To test the reproducibility of our findings between different statistical methods, we also chose the non-parametric permutation test and presented the results and brief discussion in Tables S6, S7 and Text S3 (Supplementary Materials).

## Results

### Global Measures of the Human Brain Anatomical Networks

We calculated the values of 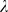 and 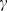 for the human brain anatomical networks derived from the conventional DTI and FLAIR-DTI datasets. Both anatomical networks showed 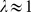 and 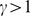, which indicated that the anatomical networks for both types of DTI datasets had small-world properties. Table 1 lists five other global parameters (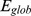, 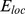, 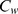, 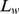 and *S*) of the anatomical networks as well as results of the statistical comparisons. Compared with the anatomical network from the conventional DTI datasets, we found statistically significant higher values of 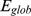 and 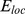 as well as of *S* but a statistically significant lower value of 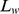 for the anatomical networks from the FLAIR-DTI datasets.

**Table 1:** Comparison between the topological properties of the human brain anatomical networks derived from the conventional DTI and FLAIR-DTI datasets.

Global parameters	Conventional DTI (Mean ± SD)	FLAIR-DTI (Mean ± SD)	*t*-value	*p*-value
*E_glob_*	0.679±0.063	0.737±0.062	−2.634	0.016*
*E_loc_*	0.986±0.076	1.064±0.082	−2.354	0.029*
*C_w_*	0.339±0.018	0.352±0.019	−1.535	0.140
*L_w_*	1.486±0.151	1.366±0.107	2.510	0.021*
*S*	0.145±0.014	0.159±0.013	−2.255	0.036*

The negative (positive) *t*-value indicates that the value of the global parameter corresponding to the FLAIR-DTI datasets is higher (lower) than that of the conventional DTI datasets. The symbol (*) stands for statistically significant difference, as determined by a threshold of *p*<0.05.

### Regional Measures of Human Brain Anatomical Networks

**Figure 2:**
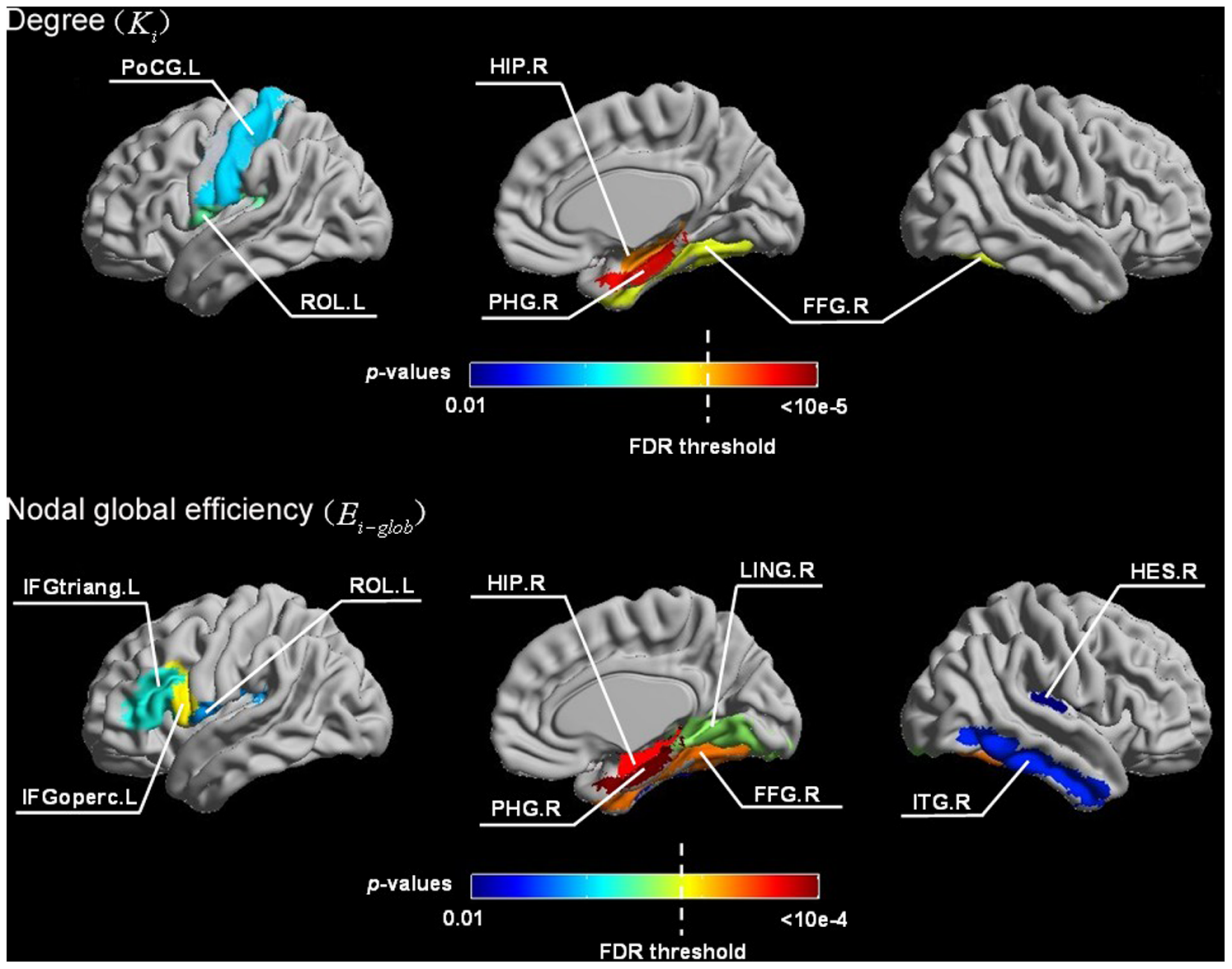
Locations of the cortical regions showing statistically significant differences in two nodal parameters, 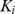 and 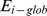, of the anatomical networks. The lower panel shows the differences in the nodal global parameter (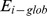), and the upper panel shows the differences in degree (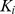) of the anatomical networks. The dotted white line shows the critical FDR threshold (*q* = 0.05; see Materials and Methods). Most of the significantly different regions were in the brain medial plane. In these regions, the values of these two nodal parameters were higher in the anatomical networks derived from the FLAIR-DTI datasets than those derived from the conventional DTI datasets.

Statistical analyses revealed no significant differences in 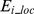 and 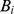, but significant differences in 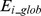 and 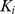. Table 2 lists the statistical comparisons between the nodal parameters of the anatomical networks corresponding to the two types of DTI datasets. Statistically significant differences in two regional nodal parameters (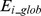
and 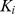) were found primarily at the same nodes, except that the number of nodes showing a difference in 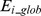 was greater than the number of nodes showing difference in 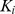 (Table 2, Fig. 2). Compared with the network corresponding to the conventional DTI datasets, the network from the FLAIR-DTI datasets showed a statistically significant higher value of degree 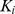 at five different brain regions, including two paralimbic regions (HIP.R and PHG.R) which survived using a height FDR correction, and three other regions (PoCG.L, FFG.R, and ROL.L) which survived using a extent threshold (*p*<0.01, uncorrected). For the nodal global efficiency (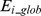), the network corresponding to the FLAIR-DTI datasets showed significantly higher values for nine different brain regions, including four regions (IFGoperc.L, HIP.R, PHG.R, and FFG.R) which survived by applying a height FDR correction and another five regions (IFGtriang.L, ROL.L, LING.R, HES.R, andITG.R) which survived using an extent threshold (*p*<0.01, uncorrected). From Table 2 and Fig. 2, we can see that at PHG.R and HIP.R, the anatomical networks showed significant differences in both the nodal parameters, 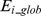 and 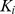.

**Table 2:** Statistically significant differences in the nodal parameters of the anatomical networks between the conventional DTI (C-DTI) and FLAIR-DTI (F-DTI) datasets.

Regions Classification		*t*-value (C-DTI) - (F-DTI)		*p-*value	
		*K_i_*	*E_i-glob_*	*K_i_*	*E_i-glob_*
***FFG.R***	Association	−3.310	−3.698	0.004*	0.001**
***HIP.R***	Subcortical	−3.909	−3.987	8.857e-4**	7.247e-4**
HES.R	Primary	–	−2.906	–	0.009*
IFGoperc.L	Association	–	−3.536	–	0.002**
IFGtriang.L	Association	–	−3.311	–	0.004*
ITG.R	Association	–	−2.940	–	0.008*
LING.R	Association	–	−3.334	–	0.003
***PHG.R***	Paralimbic	−4.919	−4.385	8.284e-5**	2.863e-4**
PoCG.L	Primary	−2.972	–	0.008*	–
***ROL.L***	Association	−3.042	−3.142	0.006*	0.005*

Note: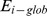 and 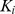 represent nodal global efficiency and degree, respectively. Bold, italic text indicates that these common brain regions showed statistically significant differences in the anatomical networks corresponding to the two types of DTI datasets with respect to both the parameters, 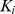 and 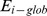. A negative *t*-value indicates that the value of the nodal parameter corresponding to the FLAIR-DTI datasets is higher than that of the conventional DTI dataset. The symbol “–” shows that these regions were not statistically significantly different with respect to 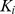 or 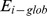.

* *p*<0.01 (uncorrected),

** *p*<0.05 (FDR corrected).

### Hub Regions of the Human Brain Anatomical Networks

**Figure 3:**
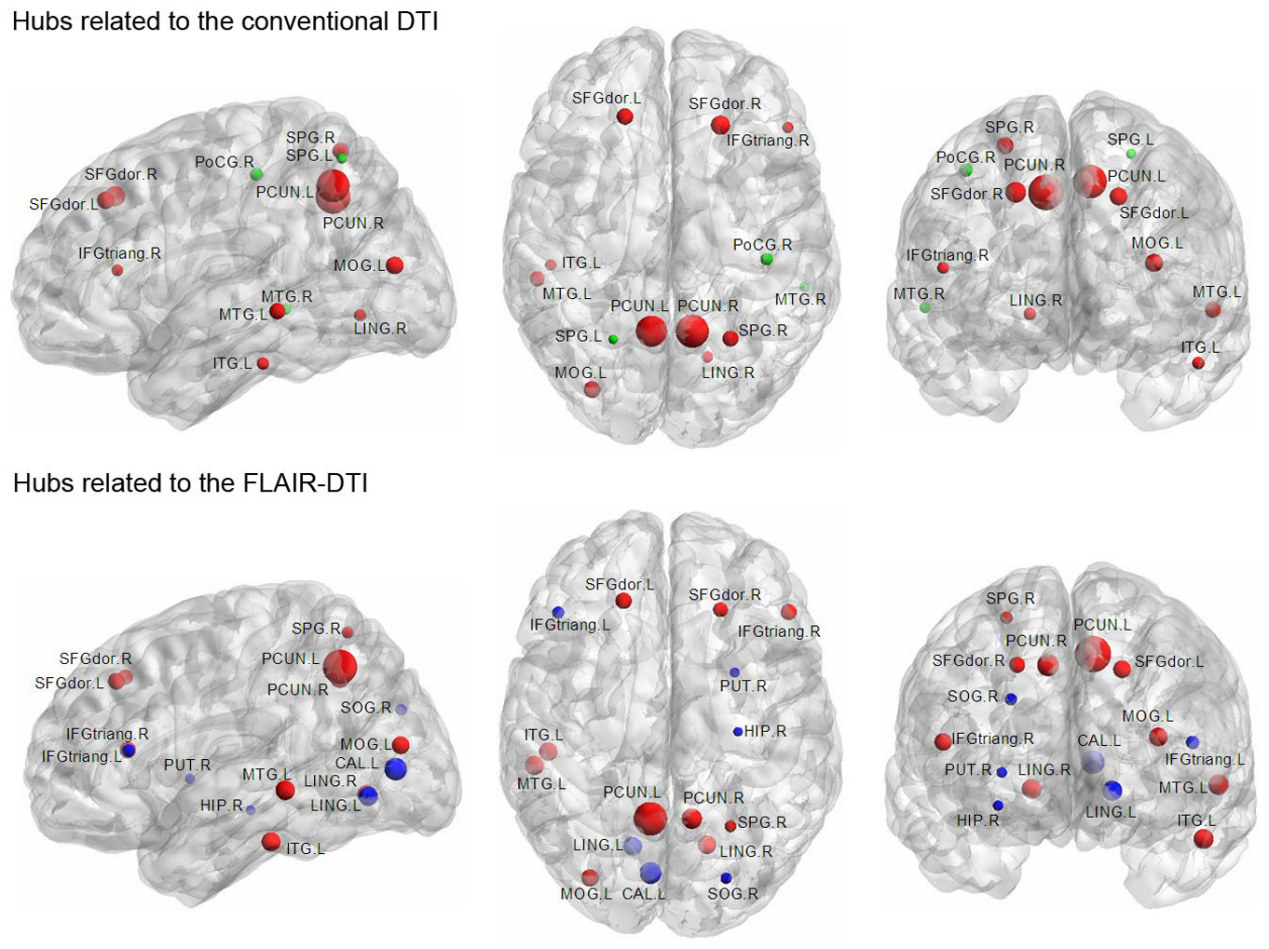
Rendering plot of the hub regions detected in the human brain anatomical networks for the conventional DTI (upper) and for the FLAIR-DTI (lower). Sixteen hub regions were identified in the anatomical network derived from the FLAIR-DTI datasets, whereas thirteen hub regions were derived in the network from the conventional DTI datasets. The size of the node represents the magnitude of the normalized betweenness centrality (see Table 3 for more details). Nodes in red represent hub regions shared by the networks derived from both types of DTI datasets. Nodes in green (blue) represent the hub regions specific to the network derived from the conventional DTI (FLAIR-DTI) datasets.

Table 3 lists the thirteen and sixteen hubs that were identified in the human brain anatomical networks corresponding to the conventional DTI datasets and FLAIR-DTI datasets, respectively. The FLAIR-DTI and conventional DTI techniques were consistent with each other for most of the identified hubs. As shown in Table 3, the hubs that were shared by the networks derived from both the conventional DTI and FLAIR-DTI datasets were the following ten association brain regions: three frontal regions (IFGtriang.R and bilateral SFGdor), four occipital regions (LING.R, MOG.L, bilateral PCUN), two temporal regions (MTG.L and ITG.L), and one parietal region (SPG.R). Moreover, the PoCG.R, SPG.L, and MTG.R were detected as hubs in the network related to the conventional DTI but not in the hubs related to the FLAIR-DTI. However, the following six brain regions, the IFGtriang.L, HIP.R, CAL.L, LING.L, SOG.R, and PUT.R, were detected as hubs in the networks related to the FLAIR-DTI datasets but not to the conventional DTI datasets. All of these brain regions are shown in Fig. 3.

**Table 3:** Hub regions of the human brain anatomical networks derived from both the conventional DTI (C-DTI) and FLAIR-DTI (F-DTI) datasets.

Regions Location		Hubs		*b_i_*		Identified as a hub in previous studies	
		C-DTI	F-DTI	C-DTI	F-DTI	Anatomical networks	Functional networks
CAL.L	Primary	N	Y	–	3.133	3,9	1
HIP.R	Subcortical	N	Y	–	2.004	–	–
IFGtriang.L	Association	N	Y	–	2.254	2,11	1
IFGtriang.R**^a^**	Association	Y	Y	2.041	2.598	2,8	1
ITG.L**^a^**	Association	Y	Y	2.095	2.818	9,10	1
LING.L	Association	N	Y	–	2.836		1,10
LING.R**^a^**	Association	Y	Y	2.114	2.814	9	1
MOG.L**^a^**	Association	Y	Y	2.574	2.670	4,5,7,8,9,11	1,10
MTG.L**^a^**	Association	Y	Y	2.434	2.865	2,11	1
MTG.R**^b^**	Association	Y	N	1.970	–	3,9,11	1,10
PCUN.L**^a^**	Association	Y	Y	3.926	4.183	3,4,5,7,8,9	1
PCUN.R**^a^**	Association	Y	Y	4.095	2.981	3,4,5,7,8,9	1
PoCG.R**^b^**	Primary	Y	N	2.178	–	2,7	1
PUT.R	Subcortical	N	Y	–	2.027	4,6,7,8	–
SFGdor.L**^a^**	Association	Y	Y	2.547	2.622	2,3, 4,5,7,9,11	1,10
SFGdor.R**^a^**	Association	Y	Y	2.806	2.477	2,3,4,5,7,8,9,11	1,6,10
SOG.R	Association	N	Y	–	2.163	2,5,6,8	1
SPG.L**^b^**	Association	Y	N	1.896	–	4	1
SPG.R**^a^**	Association	Y	Y	2.509	2.196	4,6,7,8	1

1. Achard et al 2006.

2. He et al 2007.

3. Hagmann et al 2008.

4. Iturria-Medina et al 2008.

5. Gong et al 2009.

6. He et al 2009.

7. Li et al 2009.

8. Shu et al 2009.

9. Yan et al 2010.

10. Tian et al 2011.

11. Chen et al 2008.

Note: **^a^**The hub regions shared by the networks derived from both types of DTI datasets. **^b^**The hub regions detected only from the conventional DTI datasets. The remaining six regions are the hubs detected only from the FLAIR-DTI datasets. The symbol “–” stands for “not reported” in these previous studies. “Y” indicates that the region has been identified as a “hub”, and “N” indicates that it has not been identified as a hub.

### Similarity and Variability of Nodal Parameters

**Figure 4:**
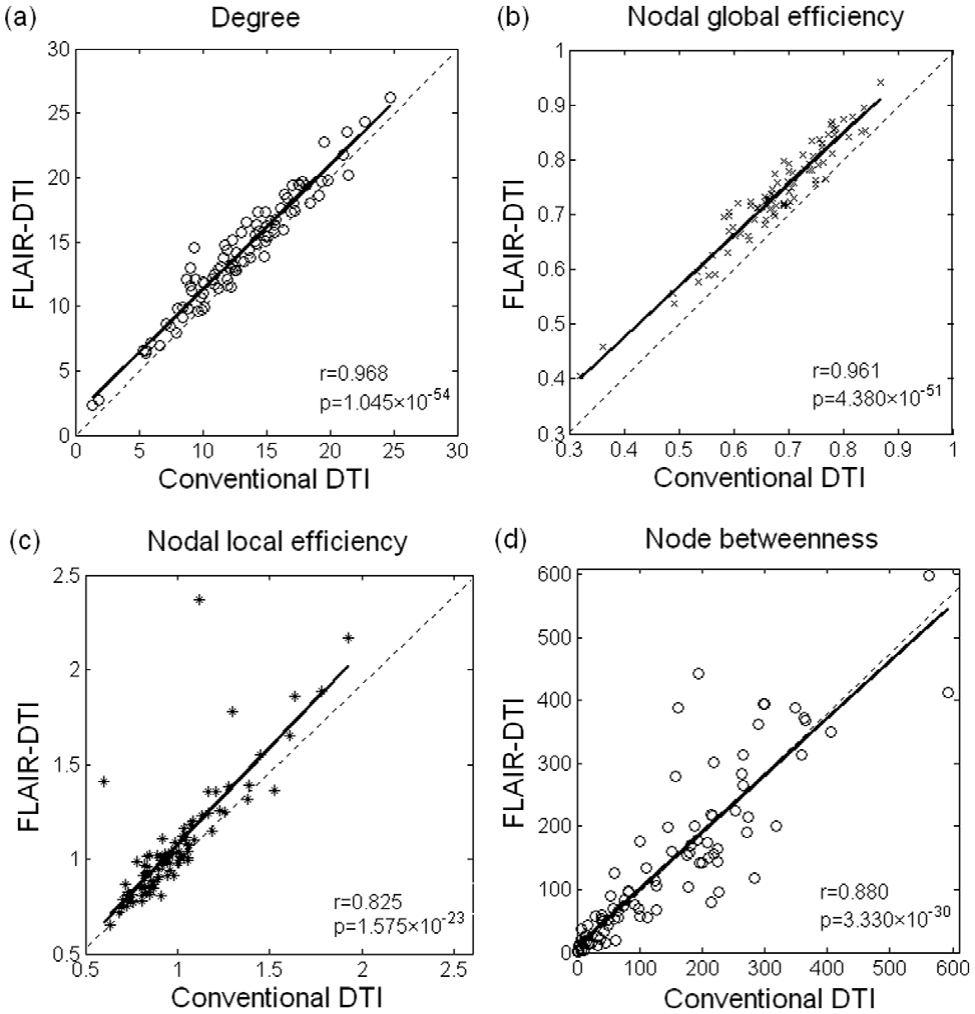
Variation in the nodal parameters derived from the conventional DTI and FLAIR-DTI datasets. The solid line represents the result of linear fitting. The ○, ×, *, and ◊ symbols in (a), (b), (c), and (d) represent the 90 brain regions from the AAL template. Three outliers in (c) correspond to HES.L, HES.R and PAL.L. Four outliers in (d) correspond to CAL.L, LING.L, PCUN.R and MTG.R.

We first calculated each regional parameter for a subject and then averaged the regional parameter across all subjects. Thus, for the four regional parameters (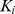,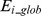,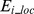, and 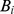), we obtained four group-averaged regional parameters. Fig. 4 shows a plot of the coincidence of the four group-averaged regional parameters (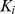,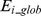,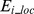, and 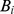) of the anatomical networks between the two types of DTI datasets. A significant correlation was observed in each of the four regional parameters between the anatomical networks corresponding to the conventional DTI and the FLAIR-DTI datasets, indicating a good agreement between the parameters derived from the two types of DTI datasets for each of these four regional parameters. We noticed that the correlation coefficient between the degree (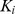) of the anatomical networks related to the two types of DTI datasets as well as the correlations between the regional global efficiency (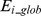) were higher than those of the regional local efficiency (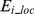) and the node betweenness (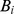) (Fig. 4). Moreover, the values of the degree (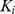) and nodal global efficiency (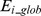) for the 90 brain regions corresponding to the FLAIR-DTI were consistently higher than those corresponding to the conventional DTI (Fig. 4a−b). The values of nodal local efficiency (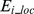) for most of the 90 brain regions corresponding to the FLAIR-DTI were higher than those corresponding to the conventional DTI and were concentrated in a small range (Fig. 4c). In addition, the patterns of the node betweenness derived from the both DTI datasets were similar to each other (Fig. 4d).

### Backbone Network

**Figure 5:**
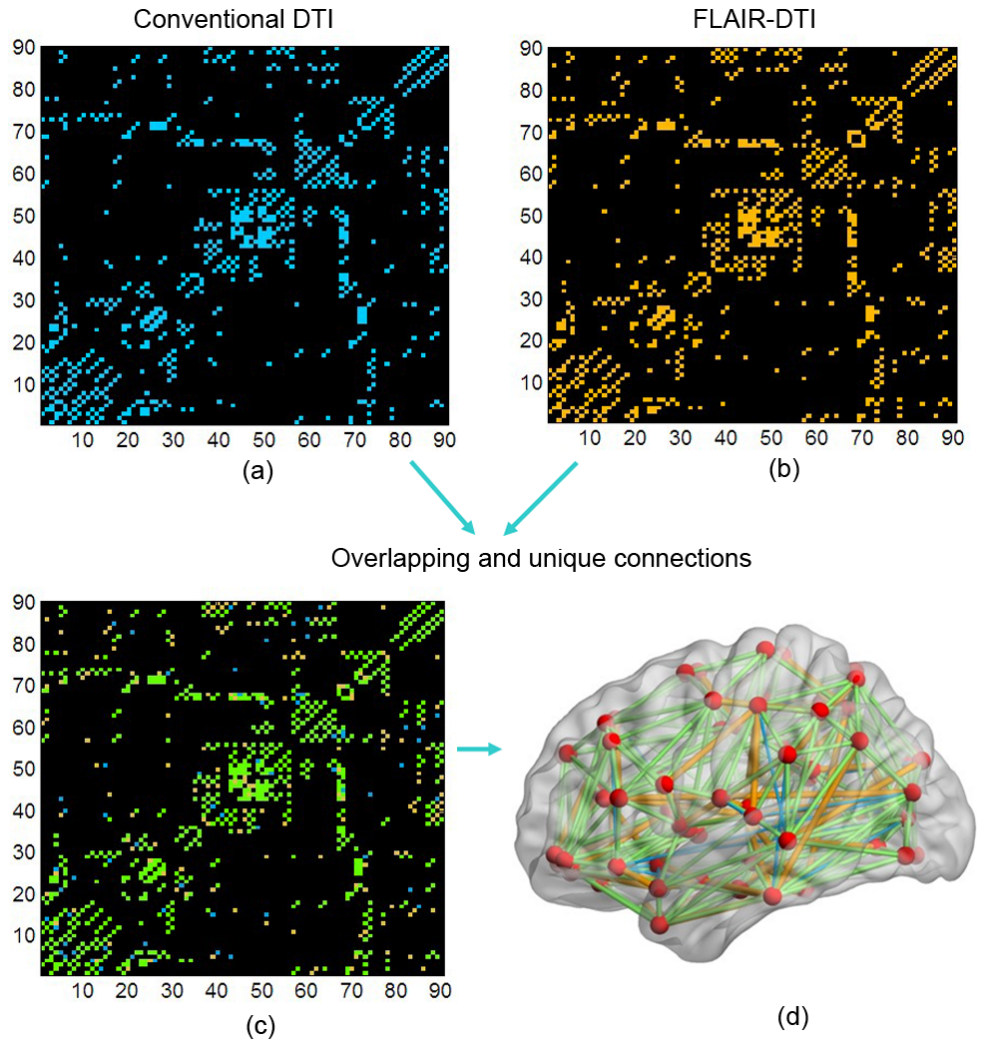
Backbone connectivity matrices of the human brain anatomical networks from the conventional DTI and FLAIR-DTI datasets. (a) For the conventional DTI datasets. (b) For the FLAIR-DTI datasets. (c) Overlapping results of the backbone networks for the conventional DTI and FLAIR-DTI datasets. Matrix elements in blue (yellow) represent the connections of the backbone network for the conventional DTI (FLAIR-DTI) datasets. Elements in green represent the common connections shared in the two backbone networks. (d) Rendering plot of edges on a cortex diagram to highlight the commonalities and differences between the edges in the two networks. Color scheme is the same as (c) but indicates edges. The numbers indicate the 90 brain regions (see Supplementary Materials Table S2 for more detail).

**Figure 6:**
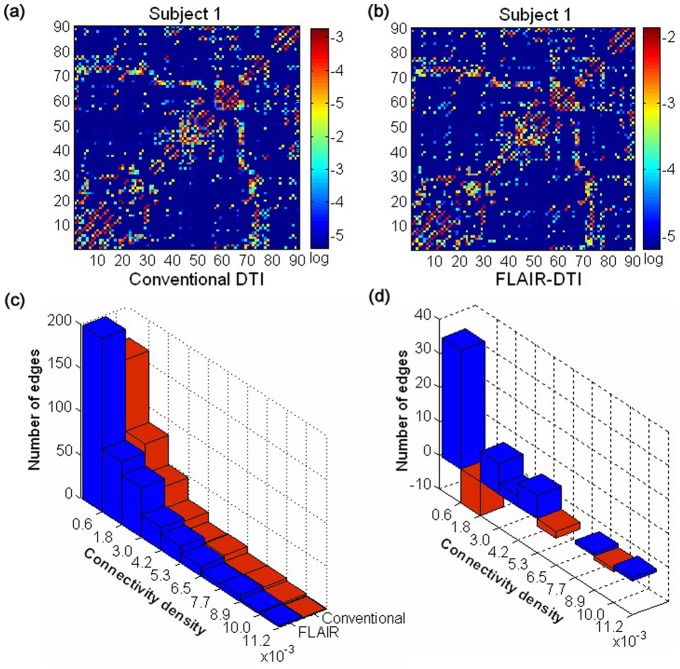
Variability in the connectivity patterns of the anatomical networks corresponding to the conventional DTI datasets and the FLAIR-DTI datasets. (a) The weighted connectivity matrix constructed from the conventional DTI data for a single subject. The weighted connectivity matrix is displayed using a logarithmic color map. (b) Same as (a) but showing the FLAIR-DTI data from a single subject. (c) Histograms of the edges derived from the backbone anatomical networks corresponding to the two types of DTI datasets. (d) Difference between the two types of DTI-based networks in the number of edges vs. connectivity density. Bars in blue (red) indicate the number of edges in the anatomical networks of the FLAIR-DTI datasets that were higher (lower) than those of the conventional DTI datasets.

The population-based backbone networks for both types of DTI datasets are shown in Fig. 5. We can see that the binary matrices of the two backbone networks have similar connectivity patterns, but the network corresponding to the conventional DTI datasets was sparser than the one corresponding to the FLAIR-DTI datasets. The sparsity of the backbone network corresponding to the FLAIR-DTI datasets was 0.1251, whereas the sparsity of the backbone network corresponding to the conventional DTI datasets was 0.1159, a decrease of 7.4% compared with that of the FLAIR-DTI dataset. We detected 464 edges in the backbone network derived from the conventional DTI datasets and 501 edges in the backbone network derived from the FLAIR-DTI datasets. These results also suggested that more connections could be detected in the backbone network from the FLAIR-DTI technique than in the one from the conventional DTI technique (Fig. 6).

### Variability in the Connectivity Patterns of the Anatomical Networks

We compared the number of edges in the networks derived from the FLAIR-DTI and conventional DTI datasets. We found that the connectivity patterns of the anatomical networks derived from the two types of DTI datasets were similar to each other. However, we noticed more edges in the anatomical networks corresponding to the FLAIR-DTI datasets. Figs. 6a–b illustrate this by showing the plots of the connectivity patterns of the anatomical networks that correspond to the two DTI datasets for a single subject. These figures show that the weighted anatomical networks that were derived from the FLAIR-DTI datasets contained more edges with high weights than those from the conventional DTI datasets (Figs. 6a–b). Based on the backbone networks, we plotted a histogram of the edges in the backbone networks that corresponded to each of the two DTI datasets (Figs. 6c–d). We found that the number of edges in the backbone network from the FLAIR-DTI was larger than the number of edges from the conventional DTI over a wide range of connectivity densities. We performed a paired *t*-test (with Bonferroni correction) to detect any statistically significant difference between the edges in the anatomical networks corresponding to the FLAIR-DTI and conventional DTI datasets, but no statistically significant difference was found.

## Discussion

In this study, we compared the topological properties of human brain anatomical networks constructed using graph theory and utilizing datasets collected by conventional DTI and FLAIR-DTI techniques. The anatomical network derived from the FLAIR-DTI datasets showed statistically significant high global efficiency (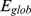) as well as high local efficiency (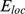). A statistical analysis indicated that the topological properties of several brain regions close to CSF-filled spaces, primarily in the periventricular regions, postcentral gyrus, inferior frontal and temporal gyri, and the regions in the visual cortex, showed significant differences between the anatomical networks derived from the FLAIR-DTI and conventional DTI datasets.

### Increased Efficiency of the Anatomical Networks Derived from the FLAIR-DTI Datasets

Our study showed that the human brain anatomical networks derived from both types of DTI datasets had almost identical path lengths (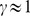) but were more locally clustered (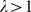) than matched random networks. This result was consistent with previous studies [6], [9], [42], [45], [56] which demonstrated that small-world topology is the conserved architecture of human brain anatomical networks.

We found the anatomical networks corresponding to the FLAIR-DTI showed a higher global and local efficiency (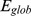,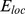) and a higher sparsity (*S*), as well as shorter characteristic path lengths (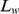) than those derived from the conventional DTI datasets (Table 1). This result is consistent with several previous studies [25], [27], [57]. Suppressing the CSF contamination may underestimate the FA value [23], [57] but improve the tractography results in periventricular regions by detecting an average of 17% more fibers in a volume than the conventional DTI technique [25]. In the present study, we found 43 more edges in the backbone network derived from the FLAIR-DTI datasets than in the one derived from the conventional DTI datasets (Fig. 6). The increased number of fiber bundles may reflect both the long-range and short-range connections in the anatomical networks from the FLAIR-DTI datasets and result in increased global and local efficiency in the anatomical networks derived from the FLAIR-DTI dataset.

One issue is that the volume of fibers detected using the FLAIR-DTI technique would be expected to contain some minor false tracts because of the lower SNR [25]. The SNR is known to affect estimated DTI measures [41] and increase the uncertainty in the direction of the major eigenvector of the diffusion tensor [34], [35]. To control the SNR effect on the estimated DTI measures and to estimate the pure CSF effect on the properties of the anatomical networks, the SNR was regressed out as a covariate in the present study. Thus, it is reasonable to believe that the increased global and local efficiency of the anatomical networks for the FLAIR-DTI datasets was primarily due to the elimination of the CSF-based partial volume effects rather than to a decreased SNR.

### Consistent Increases in Nodal Measures Observed in the Anatomical Networks Derived from the FLAIR-DTI Datasets

Increased nodal measures were observed in the anatomical network derived from the FLAIR-DTI datasets. We detected a significantly increased nodal degree (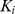) in five brain regions (HIP.R, PHG.R, PoCG.L, FFG.R and ROL.L) in the anatomical networks derived from the FLAIR-DTI datasets (Table 2 and Fig. 2). This suggests that the number of fiber tracts connecting pairs of brain regions detected from the FLAIR-DTI datasets exceeded the number from the conventional DTI datasets. In addition, we also detected significantly increased nodal global efficiency (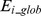) in nine brain regions (IFGoperc.L, HIP.R, PHG.R, FFG.R, IFGtriang.L, ROL.L, LING.R, HES.R and ITG.R) in the anatomical networks derived from the FLAIR-DTI datasets (Table 2 and Fig. 2). These results indicate that CSF contamination has more influence on nodal global efficiency (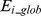) than on degree (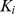).

The brain regions showed statistically significant differences in nodal degree (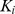) and nodal global efficiency (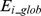) between the anatomical networks related to the two types of DTI datasets. This finding is consistent with several previous studies [21], [27]. Koo et al [58] investigated the influence of CSF contamination on gray matter mean diffusivity and found that the greatest measurement bias in the spatial pattern of the GM mean diffusivity was primarily located in the superior part of the central sulcus, the parahippocampal gyrus, and the medial part of the visual cortex. Indeed, the PHG.R and HIP.R were the two regions that showed significant differences in both the nodal parameters, 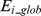 and 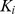, in the present study (Table 2). Both these regions are close to CSF-filled spaces (i.e., the ventricles).Previous studies have suggested that CSF contamination could lead to overestimating of the apparent diffusion coefficient (ADC) by about 15–30% [21] and to underestimating the diffusive anisotropy [23], [57] in CSF-filled spaces, such as in the periventricular regions and the brain sulci. Additionally, fiber tracking in the fornix and cingulum was also substantially improved by using the FLAIR-DTI technique [59]. These studies provide rich evidence for the increased nodal parameters (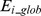,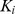) in the PHG and HIP regions in the anatomical network derived from FLAIR-DTI datasets.

Table 2 shows the regions that showed significant differences in the nodal parameter 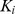 at the post postcentral gyrus (PoCG.L) and the medial part of the visual cortex (FFG.R, LING.R). These three regions are adjacent to CSF-filled regions. Fig. 2 indicates that several regions (HIP.R, PHG.R, ING.R, FFG.R, and ITG.R) were gathered into one cluster. This seems to indicate that the nodal parameters in these regions are heavily affected by CSF contamination.

In addition, we found that the nodal parameter 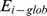 showed significant differences at the inferior frontal (IFGtriang.L, IFGoperc.L) and temporal gyri (ITG.R, HES.R) between the two anatomical networks derived from the conventional DTI datasets and FLAIR-DTI datasets (Table 2). Our findings were consistent with a previous study in which Koo et al. [58]reported that CSF contamination caused 30% of the signals to be biased in the lateral part of the prefrontal region and the temporal pole.

### More Hubs Detected in the Network Derived from the FLAIR-DTI Datasets

We identified sixteen hubs in the anatomical network from the FLAIR-DTI datasets and thirteen hubs from the conventional datasets. Among these, ten common hubs (SFGdor, IFGtriang.R, LING.R, MOG.L, SPG.R, PCUN, MTG.L, and ITG.L) were detected from the anatomical networks corresponding to both types of DTI datasets. The spatial pattern of the hubs detected from the anatomical networks corresponding to both the types of DTI datasets was consistent with the similarity of the betweenness nodes derived from both types of DTI datasets (Fig. 4d). The additional identified hubs were six regions (IFGtriang.L, HIP.R, CAL.L, LING.L, SOG.R, and PUT.R) specific to the FLAIR-DTI datasets and three regions (PoCG.R, SPG.L, MTG.R) specific to the conventional DTI datasets. Because these discrepancy hubs are located in brain regions close to CSF-filled spaces, we assume that the discrepancies between the identified hubs from the two DTI datasets may have resulted from the influences of CSF contamination, which, in turn, resulted in tractography bias.

In order to compare the identified hubs in the present study with other studies, we also listed network hubs reported in previous studies (Table 3). From Table 3, we can see that the hubs identified in both types of DTI datasets were consistent with most previous studies [11], [42], [45], [49], [60].

### Methodological Issues

Our results showed that the FLAIR-DTI technique is superior to the conventional DTI technique in global and local network efficiency metrics, especially in CSF-filled regions. However, several issues need to be addressed in the present study. First, the lower SNR and the longer scan time required for the FLAIR-DTI technique are drawbacks that need to be considered. The longer scan time for the FLAIR-DTI technique was not an important issue in this study because the subjects enrolled were healthy and cooperative during image acquisition. We controlled the SNR and found effects of CSF contamination on the topological properties of the anatomical networks. Therefore, the increased scan time was tolerable and the use of FLAIR is recommended for studying human brain anatomical networks, especially in normal healthy people. Actually, two kinds of approaches have been proposed to mitigate CSF contamination, one is the FLAIR-DTI technique and the other is the two tensor model. The two tensor model uses a data postprocessing approach to remove the CSF signal, while the FLAIR-DTI is a MRI technique to obtain the signals free from CSF contamination. Although the FLAIR-DTI technique may have suffered from disadvantages arising from a lower SNR and a longer scanning time, in this study we were able to find the effects of CSF on the properties of the anatomical network.

At present, a growing number of DW-MRI strategies have been developed for measuring and interpreting complex diffusion properties of brain white matter. The methods vary in their acquisition sampling and analysis approaches [18], including Diffusion Spectrum Imaging (DSI) [61], [62], High Angular Resolution Diffusion Imaging (HARDI), Combined Hindered and Restricted Model of Diffusion (CHARMED) imaging [63], [64], and Generalized DTI (GDTI) [65], [66], [67]. DSI is an model-free imaging approach or a directly calculating the Fourier transform approach that has the ability to map complex fiber architecture at the scale of single MRI voxels [45], [61], [62], [68], but the DSI requires a very large number of gradients (256 or 512 directions). HARDI requires a moderate amount (from about 60 to a few hundred) of diffusion gradients in a sphere of given radius [69] and transforms the data to a certain probability function (Orientation Distribution Function, or Fiber Orientation, or Probability Function in a given voxel) for estimating the apparent diffusion coefficient (ADC) profile versus diffusion gradient encoding angle [70]. There are a variety of HARDI methods such as the Q-ball imaging (QBI) [71], [72], Spherical Deconvolution (SD) [73], [74], [75], Diffusion Orientation Transform (DOT) [76], [77], and Persistent Angular Structure MRI (PAS-MRI) [69], [78] approaches. CHARMED is a *q*-space derived model to describe hindered and restricted (fast/slow) diffusion in brain tissue. GDTI uses higher order tensor model, such as 4th order tensor, to analyses the data. Although these DW-MRI strategies can deal with the crossing tracts problem in the low spatial resolution (2 mm isotropic) diffusion data in some degree, none of them has the capacity to distinguish the fibers crossing, twisting or kissing within a voxel, to determine exactly origins and terminations of fibers within the gray matter of a cortical area, and to distinguish efferent/afferent fibers or mixed projections. The single tensor model is a basic one to describe the DW-MRI signal behavior for low values of diffusion weighting (e.g., *b*<1500 s/mm^2^). It does not appear to be consistently accurate in describing the signal behavior for higher values of diffusion-weighting (e.g., *b*>2000 s/mm^2^).The single tensor model is still the basic one to process DTI data and is known to have problems with both CSF and crossing tracts [79], and FLAIR-DTI is an optimized technique to suppress the CSF contamination [80].

Second, the tractography method also influences the accuracy of the calculated network properties. We only selected the FACT algorithm [44], a deterministic tractographic method, to perform fiber tracking by setting the termination condition as FA<0.20 and a tract turning-angle>45°. Surely, the tractography and the property of the anatomical networks may be influenced by the choice of different tracking algorithms [81] and different tracking thresholds (e.g. FA = 0.25 or 0.30 with turning-angle 45° to 60°) [82]. Considering the deterministic tractography is widely used in constructing the white matter structural networks, we explored the CSF influence on the property of anatomical network constructed using deterministic tractography with the single tensor model.

Third, no ground truth exists for which anatomical network is in fact “true”. No definitive answer exists about how to select the nodes in constructing human brain anatomical networks. A study has verified that selecting different brain templates to define network nodes influences the topological properties of the brain networks [10]. Although the AAL template is widely used to define the nodes of brain networks [8], [42], many other templates have also been used, including the Brodmann atlas [9], the ANIMAL (automated nonlinear image matching and anatomical labeling) atlas [83], the LPBA40-atlas (LONI Probabilistic Brain Atlas) [16], the Harvard–Oxford Atlas [16], and the parcellation obtained using Freesurfer [45]. Moreover, no unique definition exists about how to define an edge to construct a human brain anatomical network. Various studies have defined edges as the number of fibers [14], the mean FA values of the connected fibers [52], and the weighted fiber density [42], [45], [84]. Different definitions of network nodes and edges could affect the topological properties of human brain anatomical networks. Last but not the least, we only investigated how the CSF influences the global and nodal parameters of brain anatomical networks in a single tensor model. An alternative solution would be to use a two compartment tensor model, in which one compartment models the properties of the CSF (isotropic) and the other compartment is modeled as a tensor, to process conventional DTI datasets [31], [32], [33]. The function of the two tensor model in data post-processing is equivalent to that of the FLAIR-DTI technique which can eliminate the CSF contamination in the single tensor model. Finding whether brain anatomical networks constructed from conventional DTI datasets using a two compartment tensor model are consistent with anatomical networks constructed from FLAIR-DTI datasets using a single tensor model would be revealing.

### Conclusions

Using graph theoretical approaches, we explored the influence of CSF on human brain anatomical networks. Our study suggests that human brain anatomical networks derived from FLAIR-DTI datasets have higher network efficiency than those derived from conventional DTI datasets. CSF contamination influences not only the nodal properties of the brain regions close to the CSF-filled space but also the number and location of the identified hub regions in human brain anatomical networks. In addition, CSF contamination influences the backbone network derived from conventional DTI datasets. Because human brain anatomical networks have previously been constructed primarily from diffusion datasets acquired using the conventional DTI technique with the single tensor model, our findings may have implications for human brain anatomical networks and tractographic methods in studies of normal brain development and clinical applications. In order to pinpoint the differences in human brain anatomical networks between different study groups, we suggest that, if possible, selecting CSF suppression techniques such as the FLAIR-DTI sequence to eliminate the CSF signal will increase the accuracy of human brain anatomical networks.

## Supporting Information

Figure S1
**Normal probability plot of the global parameters (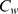, 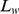).**
(TIF)

Figure S2
**Normal probability plot of the global parameters (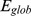,** ***S*** **).**
(TIF)

Table S1 **The signal-to-noise ratio (SNR) for the selected ROIs, fornix and splenium of the callosum (SCC), for each subject.**
(DOC)

Table S2 **Regions of the automated anatomical labeling (AAL) template and the corresponding abbreviations used in this study.**
(DOC)

Table S3 **Power analysis and normality test for different global parameters.** Note: The bold digital number means that the value of the global parameters does not obey the normal distribution.
(DOC)

Table S4 **Power analysis for nodal parameters in significant different regions.**
(DOC)

Table S5
**Normality test for the nodal parameters in the significant different regions.** Note: The bold digital number means that the value of the nodal parameter (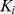 or 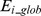) in the region does not obey the normal distribution.(DOC)

Table S6 **Global parameters of the brain anatomical networks derived from the conventional DTI and FLAIR-DTI datasets using the nonparametric permutation test.**
(DOC)

Table S7
**Statistically significant differences in the nodal parameters of the brain anatomical networks using the nonparametric permutation test.** Note: Bold text indicates the brain regions showing significant differences in both nodal parameters, 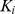 and 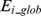, between the anatomical networks corresponding to the two types of DTI datasets.(DOC)

Text S1 **Definitions of nodal parameters and global parameters.**
(DOC)

Text S2 **Power analysis and normality test.**
(DOC)

Text S3 **Non-parametric permutation test.**
(DOC)

Text S4 **Results from the non-parametric permutation test.**
(DOC)
