# Supplementary figures and images for: Increased Global and Local Efficiency of Human Brain Anatomical Networks Detected with FLAIR-DTI Compared to Non-FLAIR-DTI
Source: PLoS One. 2013 Aug 13;8(8):e71229. doi: 10.1371/journal.pone.0071229 (PMC3742791; doi:10.1371/journal.pone.0071229)

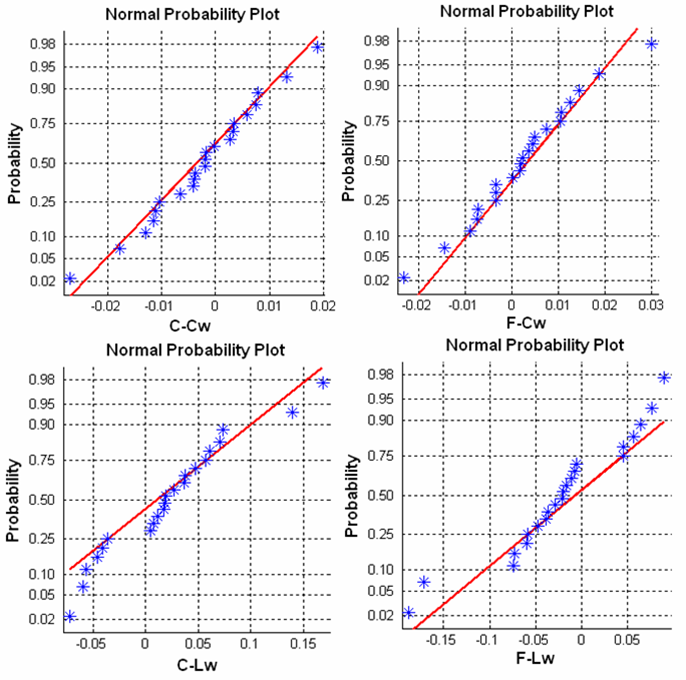

Supplement: Figure S1 — Normal probability plot of the global parameters (, ). (TIF) [file pone.0071229.s001.tif]

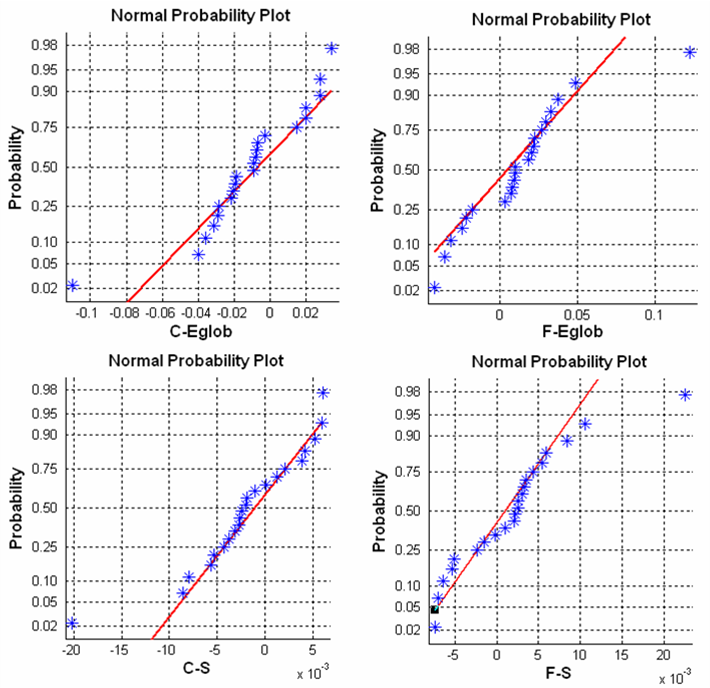

Supplement: Figure S2 — Normal probability plot of the global parameters (, S ). (TIF) [file pone.0071229.s002.tif]
