# Supplementary material for: Increased Global and Local Efficiency of Human Brain Anatomical Networks Detected with FLAIR-DTI Compared to Non-FLAIR-DTI
Source: PLoS One. 2013 Aug 13;8(8):e71229. doi: 10.1371/journal.pone.0071229 (PMC3742791; doi:10.1371/journal.pone.0071229)
Supplement: Table S1 — The signal-to-noise ratio (SNR) for the selected ROIs, fornix and splenium of the callosum (SCC), for each subject. (DOC) [file pone.0071229.s003.doc]

**Table S1**. The signal-to-noise ratio (SNR) for the selected ROIs, fornix and splenium of the callosum (SCC), for each subject. C-DTI refers to the conventional DTI scans, and F-DTI refers to the FLAIR-DTI scans.

| Subject | SNR in the fornix | | SNR in the SCC | |
| --- | --- | --- | --- | --- |
|  | C-DTI | F-DTI | C-DTI | F-DTI |
| 1 | 32.76 | 23.72 | 20.01 | 19.83 |
| 2 | 38.47 | 23.00 | 23.72 | 19.66 |
| 3 | 35.58 | 20.08 | 19.79 | 18.40 |
| 4 | 35.60 | 22.63 | 26.38 | 23.85 |
| 5 | 37.09 | 22.16 | 22.83 | 21.51 |
| 6 | 35.55 | 19.47 | 21.28 | 22.22 |
| 7 | 38.89 | 22.88 | 23.25 | 20.83 |
| 8 | 35.37 | 22.14 | 21.02 | 19.09 |
| 9 | 35.96 | 23.03 | 18.75 | 17.89 |
| 10 | 33.15 | 24.66 | 22.71 | 21.86 |
| 11 | 34.40 | 20.46 | 19.85 | 20.08 |
| 12 | 37.17 | 24.80 | 20.25 | 19.95 |
| 13 | 39.73 | 22.85 | 23.97 | 22.60 |
| 14 | 41.67 | 21.83 | 24.01 | 19.68 |
| 15 | 37.76 | 23.41 | 23.50 | 20.71 |
| 16 | 37.41 | 23.11 | 22.69 | 18.27 |
| 17 | 39.32 | 23.09 | 21.67 | 19.69 |
| 18 | 37.49 | 22.11 | 18.87 | 19.53 |
| 19 | 41.58 | 22.10 | 22.26 | 19.90 |
| 20 | 42.44 | 19.66 | 22.66 | 18.41 |
| 21 | 33.93 | 23.40 | 21.87 | 19.22 |
| 22 | 39.32 | 19.47 | 24.38 | 19.51 |
| Mean ± std | 37.30 ± 2.71 | 22.28 ± 1.56 | 22.08 ± 1.95 | 20.12 ± 1.51 |
