# Supplementary material for: Increased Global and Local Efficiency of Human Brain Anatomical Networks Detected with FLAIR-DTI Compared to Non-FLAIR-DTI
Source: PLoS One. 2013 Aug 13;8(8):e71229. doi: 10.1371/journal.pone.0071229 (PMC3742791; doi:10.1371/journal.pone.0071229)
Supplement: Table S2 — Regions of the automated anatomical labeling (AAL) template and the corresponding abbreviations used in this study. (DOC) [file pone.0071229.s004.doc]

**Table S2**. Regions of the automated anatomical labeling (AAL) template and the corresponding abbreviations used in this study. These brain regions were originally described by Tzourio-Mazoyer et al. (2002). The abbreviations are listed according to Salvador et al. (2005) and Achard et al. (2006).

| Index | Regions | Abb. | Index | Regions | Abb. |
| --- | --- | --- | --- | --- | --- |
| 1 | Precentral gyrus | PreCG | 24 | Lingual gyrus | LING |
| 2 | Superior frontal gyrus (dorsal) | SFGdor | 25 | Superior Occipital gyrus | SOG |
| 3 | Orbitofrontal cortex (superior) | ORBsup | 26 | Middle occipital gyrus | MOG |
| 4 | Middle frontal gyrus | MFG | 27 | Inferior occipital gyrus | IOG |
| 5 | Orbitofrontal cortex (middle) | ORBmid | 28 | Fusiform gyrus | FFG |
| 6 | Inferior frontal gyrus (opercular) | IFGoperc | 29 | Postcentral gyrus | PoCG |
| 7 | Inferior frontal gyrus (triangular) | IFGtriang | 30 | Superior parietal gyrus | SPG |
| 8 | Orbitofrontal cortex (inferior) | ORBinf | 31 | Inferior parietal lobule | IPL |
| 9 | Rolandic operculum | ROL | 32 | Supramarginal gyrus | SMG |
| 10 | Supplementary motor area | SMA | 33 | Angular gyrus | ANG |
| 11 | Olfactory | OLF | 34 | Precuneus | PCUN |
| 12 | Superior frontal gyrus (medial) | SFGmed | 35 | Paracentral lobule | PCL |
| 13 | Orbitofrontal cortex (medial) | ORBmed | 36 | Caudate | CAU |
| 14 | Rectus gyrus | REC | 37 | Putamen | PUT |
| 15 | Insula | INS | 38 | Pallidum | PAL |
| 16 | Anterior cingulate gyrus | ACG | 39 | Thalamus | THA |
| 17 | Middle cingulate gyrus | MCG | 40 | Heschl gyrus | HES |
| 18 | Posterior cingulate gyrus | PCG | 41 | Superior temporal gyrus | STG |
| 19 | Hippocampus | HIP | 42 | Temporal pole (superior) | TPOsup |
| 20 | Parahippocampal gyrus | PHG | 43 | Middle temporal gyrus | MTG |
| 21 | Amygdala | AMYG | 44 | Temporal pole (middle) | TPOmid |
| 22 | Calcarine cortex | CAL | 45 | Inferior temporal gyrus | ITG |
| 23 | Cuneus | CUN |  |  |  |
