# Supplementary material for: Increased Global and Local Efficiency of Human Brain Anatomical Networks Detected with FLAIR-DTI Compared to Non-FLAIR-DTI
Source: PLoS One. 2013 Aug 13;8(8):e71229. doi: 10.1371/journal.pone.0071229 (PMC3742791; doi:10.1371/journal.pone.0071229)
Supplement: Table S3 — Power analysis and normality test for different global parameters. Note: The bold digital number means that the value of the global parameters does not obey the normal distribution. (DOC) [file pone.0071229.s005.doc]

**Table S3**. Power analysis and normality test for different global parameters.

| Global parameters | Power analysis | | Normality test  (*p*-value) | |
| --- | --- | --- | --- | --- |
|  | Effect size | Statistical power | C-DTI | F-DTI |
| $E_{glob}$ | 0.952 | 0.989 | 0.344 | 0.303 |
| $E_{loc}$ | 0.886 | 0.976 | **0.033** | **0.034** |
| $C_{w}$ | 0.661 | 0.828 | > 0.5 | > 0.5 |
| $L_{w}$ | 0.940 | 0.986 | > 0.5 | 0.315 |
| *S* | 1.056 | 0.999 | 0.472 | 0.323 |

Note: The bold digital number means that the value of the global parameters does not obey the normal distribution.
