# Supplementary material for: Increased Global and Local Efficiency of Human Brain Anatomical Networks Detected with FLAIR-DTI Compared to Non-FLAIR-DTI
Source: PLoS One. 2013 Aug 13;8(8):e71229. doi: 10.1371/journal.pone.0071229 (PMC3742791; doi:10.1371/journal.pone.0071229)
Supplement: Table S4 — Power analysis for nodal parameters in significant different regions. (DOC) [file pone.0071229.s006.doc]

**Table S4**. Power analysis for nodal parameters in significant different regions.

| Regions | Power analysis | | Power analysis | |
| --- | --- | --- | --- | --- |
|  | Effect size $K_{i}$ | Statistical power $K_{i}$ | Effect size $E_{i-glob}$ | Statistical power $E_{i-glob}$ |
| FFG.R | 0.926 | 0.985 | 1.072 | 0.998 |
| HIP.R | 1.127 | 1.000 | 0.822 | 0.954 |
| HES.R | ─ | ─ | 0.821 | 0.954 |
| IFGoperc.L | ─ | ─ | 0.815 | 0.951 |
| IFGtriang.L | ─ | ─ | 0.691 | 0.862 |
| ITG.R | ─ | ─ | 0.968 | 0.991 |
| LING.R | ─ | ─ | 1.069 | 0.998 |
| PHG.R | 1.777 | 1.000 | 1.321 | 1.000 |
| PoCG.L | 0.397 | 0.397 | ─ | ─ |
| ROL.L | 0.701 | 0.871 | 0.584 | 0.752 |
