# Supplementary material for: Increased Global and Local Efficiency of Human Brain Anatomical Networks Detected with FLAIR-DTI Compared to Non-FLAIR-DTI
Source: PLoS One. 2013 Aug 13;8(8):e71229. doi: 10.1371/journal.pone.0071229 (PMC3742791; doi:10.1371/journal.pone.0071229)
Supplement: Table S5 — Normality test for the nodal parameters in the significant different regions. Note: The bold digital number means that the value of the nodal parameter ( or ) in the region does not obey the normal distribution. (DOC) [file pone.0071229.s007.doc]

**Table S5**. Normality test for the nodal parameters in the significant different regions.

| Regions | Normality test (*p*-value) | | Normality test (*p*-value) | |
| --- | --- | --- | --- | --- |
|  | C-DTI $K_{i}$ | F-DTI $K_{i}$ | C-DTI $E_{i-glob}$ | F-DTI $E_{i-glob}$ |
| FFG.R | 0.051 | 0.067 | >0.5 | **0.044** |
| HIP.R | 0.490 | **0.049** | 0.348 | 0.431 |
| HES.R | ─ | ─ | 0.374 | 0.372 |
| IFGoperc.L | ─ | ─ | 0.264 | 0.092 |
| IFGtriang.L | ─ | ─ | 0.166 | >0.5 |
| ITG.R | ─ | ─ | >0.5 | 0.255 |
| LING.R | ─ | ─ | 0.065 | 0.447 |
| PHG.R | 0.218 | >0.5 | 0.494 | >0.5 |
| PoCG.L | 0.334 | >0.5 | ─ | ─ |
| ROL.L | **0.001** | 0.116 | 0.412 | 0.060 |

Note: The bold digital number means that the value of the nodal parameter (*K*_i_ or *E*_i-glob_) in the region does not obey the normal distribution.
