# Supplementary material for: Increased Global and Local Efficiency of Human Brain Anatomical Networks Detected with FLAIR-DTI Compared to Non-FLAIR-DTI
Source: PLoS One. 2013 Aug 13;8(8):e71229. doi: 10.1371/journal.pone.0071229 (PMC3742791; doi:10.1371/journal.pone.0071229)
Supplement: Table S6 — Global parameters of the brain anatomical networks derived from the conventional DTI and FLAIR-DTI datasets using the nonparametric permutation test. (DOC) [file pone.0071229.s008.doc]

**Table S6.** Global parameters of the brain anatomical networks derived from the conventional DTI and FLAIR-DTI datasets using the nonparametric permutation test.

| Global  parameters | Conventional DTI  (Mean ± SD) | FLAIR-DTI  (Mean ± SD) | permutation  *p*-value |
| --- | --- | --- | --- |
| $E_{glob}$ | 0.679 ± 0.063 | 0.737 ± 0.062 | 0.1995 |
| $E_{loc}$ | 0.986 ± 0.076 | 1.064 ± 0.082 | 0.2317 |
| $C_{w}$ | 0.339 ± 0.018 | 0.352 ± 0.019 | 0.305 |
| $L_{w}$ | 1.486 ± 0.151 | 1.366 ± 0.107 | 0.2072 |
| *S* | 0.145 ± 0.014 | 0.159 ± 0.013 | 0.2416 |
