# Supplementary material for: Increased Global and Local Efficiency of Human Brain Anatomical Networks Detected with FLAIR-DTI Compared to Non-FLAIR-DTI
Source: PLoS One. 2013 Aug 13;8(8):e71229. doi: 10.1371/journal.pone.0071229 (PMC3742791; doi:10.1371/journal.pone.0071229)
Supplement: Table S7 — Statistically significant differences in the nodal parameters of the brain anatomical networks using the nonparametric permutation test. Note: Bold text indicates the brain regions showing significant differences in both nodal parameters, and , between the anatomical networks corresponding to the two types of DTI datasets. (DOC) [file pone.0071229.s009.doc]

**Table S7.** Statistically significant differences in the nodal parameters of the brain anatomical networks using the nonparametric permutation test.

| Regions | Classification | Permutation (*p*-value) | |
| --- | --- | --- | --- |
|  |  | $K_{i}$ | $E_{i-glob}$ |
| **FFG.R** | Association | 0.0274 | 0.0238 |
| **HIP.R** | Subcortical | 0.0181 | 0.0197 |
| HES.R | Primary | ─ | 0.0439 |
| IFGoperc.L | Association | ─ | 0.0270 |
| IFGtriang.L | Association | ─ | 0.0301 |
| ITG.R | Association | ─ | 0.0440 |
| LING.R | Association | ─ | 0.0285 |
| **PHG.R** | Paralimbic | 0.0099 | 0.0117 |
| PoCG.L | Primary | 0.0425 | ─ |
| **ROL.L** | Association | 0.0377 | 0.0366 |
| IOG.R | Association | ─ | 0.0483 |

Note: Bold text indicates the brain regions showing significant differences in both nodal parameters, *K*_i_ and *E*_i-glob_, between the anatomical networks corresponding to the two types of DTI datasets.
