# Supplementary material for: Increased Global and Local Efficiency of Human Brain Anatomical Networks Detected with FLAIR-DTI Compared to Non-FLAIR-DTI
Source: PLoS One. 2013 Aug 13;8(8):e71229. doi: 10.1371/journal.pone.0071229 (PMC3742791; doi:10.1371/journal.pone.0071229)
Supplement: Text S1 — Definitions of nodal parameters and global parameters. (DOC) [file pone.0071229.s010.doc]

**Definitions of nodal parameters**

The degree of the node $i$ is defined as the number of edges linking to that node, $K_{i}=\sum_{j\in G} a_{ij}$, where $a_{ij}$ is the $i$th row and$j$th column element of adjacency matrix. Nodal local efficiency of the node $i$ is computed as

$E_{i\_loc}=\frac{1}{N_{G_{i}}(N_{G_{i}}-1)}\sum_{i,j\in G_{i}} \frac{1}{l_{ij}}$, (1)

where $G_{i}$ is the subgraph containing the set of nodes that are the direct neighbors of the node $i$. Nodal global efficiency of the node $i$ is given by

$E_{i\_glob}=\frac{1}{N-1}\sum_{i\neq j\in G} \frac{1}{l_{ij}}$. (2)

The node betweenness describes the number of shortest paths between pairs of other nodes that pass through a given node $i$ . It is defined as

$B_{i}=\sum_{j\neq i\neq k\in G} \frac{\sigma_{jk}\left(i\right)}{\sigma_{jk}}$, (3)

where $\sigma_{jk}$ is the number of shortest paths from node $j$ to node $k$, and$\sigma_{jk}\left(i\right)$ is the number of the number of shortest paths from node $j$ to node $k$ that pass through the node $i$ within the graph G.

**Definitions of global parameters**

The network can be represented as a graph *G*(*N*, *M*) with *N* nodes and *M* edges. The weighted clustering coefficient of node $i$ is given by

$c_{i}=\frac{2}{K_{i}(K_{i}-1)}\sum_{j,k} (\tilde{w}_{ij}\tilde{w}_{jk}\tilde{w}_{ki})^{1/3}$. (4)

The mean weighted clustering coefficient of the whole anatomical network ($C_{w}=1/N\sum_{i=1,2...,N} c_{i}$) is the average of the clustering coefficients over all nodes; In addition, $\tilde{w}_{ij}$ is the weight which is scaled by the largest weight in the network, $\tilde{w}_{ij}=w_{ij}/\max(w_{ij})$. The weighted characteristic shortest path length $L_{w}$ can be calculated by using a ‘‘harmonic mean’’ length between any pair of nodes

$L_{w}=\frac{1}{\frac{1}{N(N-1)}\sum_{j\neq i\in G} \frac{1}{l_{ij}}}$, (5)

where $l_{ij}$ is the shortest path length between nodes $i$ and$j$. The global efficiency is defined as

$E_{glob}=\frac{1}{N\left(N-1\right)}\sum_{j\neq i\in G} \frac{1}{l_{ij}}$. (6)

The local efficiency of the network is measured by

$E_{loc}=\frac{1}{N}\sum_{i\in G} E_{glob}\left(G_{i}\right)$, (7)

where $E_{glob}(G_{i})$ is the global efficiency of $G_{i}$, the sub-graph composed of the neighbors of node $i$. The sparsity (*S*) of the network is defined as the total number of edges in a network divided by the maximum possible number of edges.

Reference:

1. Achard, S. and E. Bullmore, *Efficiency and cost of economical brain functional networks.* PLoS Comput Biol, 2007. **3**(2): p. 174-183.

2. Girvan, M. and M.E. Newman, *Community structure in social and biological networks.* Proc Natl Acad Sci U S A, 2002. **99**(12): p. 7821-6.

3. Onnela, J.P., et al., *Intensity and coherence of motifs in weighted complex networks.* Phys Rev E Stat Nonlin Soft Matter Phys, 2005. **71**(6 Pt 2): p. 065103.

4. Newman, M.E.J., *The structure and function of complex networks.* Siam Review, 2003. **45**(2): p. 167-256.
