# Supplementary material for: Increased Global and Local Efficiency of Human Brain Anatomical Networks Detected with FLAIR-DTI Compared to Non-FLAIR-DTI
Source: PLoS One. 2013 Aug 13;8(8):e71229. doi: 10.1371/journal.pone.0071229 (PMC3742791; doi:10.1371/journal.pone.0071229)
Supplement: Text S2 — Power analysis and normality test. (DOC) [file pone.0071229.s011.doc]

**Power analysis**

In fact, there are some discussions about the sample size in the neuroimaging studies. For example, Friston suggested 16 or more subjects should be included in a neuroimaging study to make an inference, but Poldrack et al , Kelly et al. , Ingre et al. , and Lindquist et al. emphatically outlined the need for larger sample sizes, appropriate correction for multiple comparisons and robust statistical methods.

We performed the power analysis to justify the sample size and the statistical power of our results in this study. Statistical power, $1-\beta$, indicates the probability of rejecting a false hypothesis *H*_0_ . Effect size can be used to measure the strength of the relationship between two variables and can help us to determine if the deference is real or due to a change of factors. Effect size is frequently used in [estimating sample sizes](http://en.wikipedia.org/wiki/Estimating_sample_sizes). A lower effect size indicates a necessity of larger sample sizes, and vice versa. Cohen divides effect sizes into the small, medium and large which correspond to 0.2, 0.5 and 0.8, respectively.

According to the Cohen's definition , we computed the sample size, statistical power, and effect size of the global parameters, and the results are listed in Table S3. From Table S3, we see that the effect sizes of the global parameters ($E_{glob}$, $E_{loc}$,$L_{w}$, and *S*) reached the large level except of the effect size of $C_{w}$ reaching the medium level of Cohen's standard. This indicates that the significant deference is not due to chance, and the sample size in this study is large enough to make the inference of difference. In addition, Table S3 shows that all the global parameters hold good statistical powers, the lowest of the statistical power is 0.828 and the highest is close to 1. The higher statistical power illustrates the validity of our statistical method in some degree.

Similarly, we estimated the statistical power and effect size for the nodal parameters ($K_{i}$ and $E_{i-glob}$) of the significantly different regions (Table S4). This Table shows that for the parameter $K_{i}$, the values of effect size in four of five significant different regions (FFG.R, HIP.R, PHG.R, and ROL.L) reached to the medium level except for the PoCG.L, and in other three regions (FFG.R, HIP.R, and PHG.R) were above 0.926. As for the parameter $E_{i-glob}$ (Table S4), the values of effect size in seven brain regions (FFG.R, HIP.R, PHG.R, HES.R, IFGoperc.L, ITG.R, and LING.R) achieved the large level, and in the other two regions (IFGtriang.L and ROL.L) reached the medium standard. Table S4 also shows that almost all the regions with significant different nodal parameters have higher statistical power values, except of the value of statistical power in ROL.L being 0.752. Thus, the power analysis on the global and nodal parameters indicated that the sample size of 22 subjects in our study was enough to make an inference.

**Normality test**

We performed the normality test on the residuals of the global parameters and nodal parameters by using the Lilliefors test . The results are listed in the Tables S3 and Table S5. We found that the residuals on the four global parameters ($E_{glob}$,$C_{w}$,$L_{w}$, and *S*) satisfy the normality distribution, and the nodal parameters ($K_{i}$ and $E_{i-glob}$) nearly satisfy the normality distribution. We also plot the normal probability of the global parameters for the two types of DTI datasets in Figs. S1 and S2. If the given parameter satisfies normal distribution, the plot will be linear, otherwise the plot contains curves. From Figs. S1 and S2, we can see that the plots for the four global parameters ($E_{glob}$,$C_{w}$,$L_{w}$, and *S*) in two types of DTI datasets are linear.

1. Friston, K., *Ten ironic rules for non-statistical reviewers.* Neuroimage, 2012. **61**(4): p. 1300-10.

2. Friston, K., *Sample size and the fallacies of classical inference.* NeuroImage, 2013(0).

3. Poldrack, R.A., *The future of fMRI in cognitive neuroscience.* NeuroImage, 2012. **62**(2): p. 1216-20.

4. Kelly, C., et al., *Characterizing variation in the functional connectome: promise and pitfalls.* Trends Cogn. Sci., 2012. **16**(3): p. 181-8.

5. Ingre, M., *Why small low-powered studies are worse than large high-powered studies and how to protect against “trivial” findings in research: Comment on Friston (2012).* NeuroImage, 2013(0).

6. Lindquist, M.A., B. Caffo, and C. Crainiceanu, *Ironing out the statistical wrinkles in “Ten Ironic Rules”.* NeuroImage, 2013(0).

7. Cohen, J., *A power primer.* Psychological Bulletin, 1992. **112**(1): p. 155-9.

8. Lilliefors, *On the Kolmogorov–Smirnov test for normality with mean and variance unknown.* Journal of the American Statistical Association, 1967. **62**: p. 399-402.
