# Supplementary material for: Increased Global and Local Efficiency of Human Brain Anatomical Networks Detected with FLAIR-DTI Compared to Non-FLAIR-DTI
Source: PLoS One. 2013 Aug 13;8(8):e71229. doi: 10.1371/journal.pone.0071229 (PMC3742791; doi:10.1371/journal.pone.0071229)
Supplement: Text S3 — Non-parametric permutation test. (DOC) [file pone.0071229.s012.doc]

**Non-parametric permutation** **test**

Non-parametric permutation test is widely used in previous studies to test between-group difference. Here we also used the non-parametric permutation test to determine the significant differences in network parameters between the conventional DTI and FLAIR-DTI. Briefly, each subject was randomly assigned to one of two random groups consisting of the same number of subjects in the conventional DTI and FLAIR-DTI datasets. We repeated 10,000 permutations, resulting in a sampled between-group difference in null distribution for each of graph-based metric. Finally, we assigned a *p*-value to the between-group difference by computing the proportion of the difference that exceeded the null distribution values. A significance threshold of *p* < 0.05 was used for testing each of the graph-based metrics. The results are listed in Tables S6 and S7.

From the Table S7, we can see that the numbers and locations of the significant different regions in the two nodal parameters ($K_{i}$ and $E_{i-glob}$) detected using the permutation test are nearly same as those detected using the paired *t*-test. Four regions, FFG.R, HIP.R, PHG.R, and ROL.L, showing significant differences in the two nodal parameters ($K_{i}$ and $E_{i-glob}$), were found either using the non-parametric permutation test or using the paired *t*-test. The numbers and locations of the regions showing significant different regions in the parameters $K_{i}$ and $E_{i-glob}$ are identical when using the non-parametric permutation test or using the paired *t*-test, except of one additional region (IOG.R) showing significant difference in $E_{i-glob}$ detected using the non-parametric permutation test. From Table 6, we see no significantly difference in any of the global parameters between the two types of DTI datasets. The reason may be that the significant difference detected using the parametric test, the paired *t*-test, is marginal significant (0.01 < *p* < 0.05).

1. Wang, J.H., et al., *Disrupted Functional Brain Connectome in Individuals at Risk for Alzheimer's Disease.* Biological Psychiatry, 2013. **73**(5): p. 472-481.

2. Zhang, Z., et al., *Altered functional-structural coupling of large-scale brain networks in idiopathic generalized epilepsy.* Brain, 2011. **134**(Pt 10): p. 2912-28.

3. Wang, B., et al., *Brain anatomical networks in world class gymnasts: a DTI tractography study.* Neuroimage, 2013. **65**: p. 476-87.
